# Lymphovascular invasion, race, and the 21-gene recurrence score in early estrogen receptor-positive breast cancer

**DOI:** 10.1038/s41523-021-00231-x

**Published:** 2021-03-01

**Authors:** Della Makower, Juan Lin, Xiaonan Xue, Joseph A. Sparano

**Affiliations:** 1 Montefiore Einstein Center for Cancer Care, Bronx, NY USA; 2 Albert Einstein Cancer Center, Bronx, NY USA

**Keywords:** Breast cancer, Prognostic markers, Predictive markers

## Abstract

Lymphovascular invasion (LVI) and Black race are associated with poorer prognosis in early breast cancer (EBC). We evaluated the association between LVI and race, and whether LVI adds prognostic benefit to the 21-gene recurrence score (RS) in EBC. Women with ER+ HER2− EBC measuring up to 5 cm, with 0–3 involved axillary nodes, diagnosed between 1 January 2010 and 1 January 2014, who underwent surgery as first treatment and had available RS, were identified in the NCDB database. Bivariate associations between two categorical variables were examined using chi-square test. Multivariate Cox proportional hazards model were used to assess the association of LVI, race, and other covariates with overall survival (OS). 77,425 women, 65,018 node-negative (N0), and 12,407 with 1–3 positive (N+) nodes, were included. LVI was present in 12.7%, and associated with poor grade, RS 26–100, and N+ (all *p* < 0.0001), but not Black race. In multivariate analysis, LVI was associated with worse OS in N0 [HR 1.37 (95% CI 1.27, 1.57], but not N+ EBC. LVI was associated with worse OS in N0 patients with RS 11–25 [HR 1.31 (95% CI 1.09, 1.57)] and ≥26 [HR 1.58 (95% CI 1.30, 1.93)], but not RS 0–10. No interaction between LVI and chemotherapy benefit was seen. Black race was associated with worse OS in N0 (HR 1.21, *p* = 0.009) and N+ (HR 1.37, *p* = 0.015) disease. LVI adds prognostic information in ER+, HER2−, N0 BCA with RS 11–100, but does not predict chemotherapy benefit. Black race is associated with worse OS, but not LVI.

## Introduction

Lymphovascular invasion (LVI), the presence of tumor cells within the lumen of lymphatic or vascular system in the tumor primary site, is associated with a higher risk of regional lymph node metastasis at diagnosis and subsequent distant recurrence in early breast cancer (EBC)^1–7^. Multiparameter gene expression assays also provide prognostic information for distant recurrence in hormone receptor-positive (HR+), HER2 negative breast (HER2−) cancer^8,9^. The 21-gene recurrence score (RS) assay is both prognostic for recurrence and predictive of chemotherapy benefit in EBC^10–13^, including women with up to three positive axillary nodes^14,15^. The extent to which LVI adds prognostic information or predictive information for chemotherapy benefit to the 21-gene RS or other assays has not been adequately evaluated^16^.

Black race has also been associated with worse prognosis in EBC. Although disparities in access to care play a role in Black patients’ poorer outcomes^17,18^, survival gaps are also seen for Black women receiving contemporary adjuvant therapy on NCI-sponsored clinical trials^19–22^, suggesting that factors other than care disparities contribute to their poorer prognosis. For example, in the TAILORx trial, Black race was associated with a 1.4-fold higher risk of recurrence after adjustment for clinicopathologic characteristics, despite similar chemotherapy and endocrine therapy use and similar RS distribution^23^. Thus, biological factors that are not captured by the 21-gene RS, including racial differences in the tumor microenvironment^24,25^, may contribute to the worse outcomes for Black women with EBC.

The presence of LVI represents a manifestation of an interaction between the tumor and its microenvironment. The molecular mechanisms underlying LVI are complex and not fully defined, but pathways involving cytokine-receptor interactions have been implicated^26–28^. Studies evaluating racial differences in BCA tumor microenvironment have described the differential expression of genes associated with inflammation and angiogenesis^24,25,29–31^, as well as increased microvessel density^29,31^ and increased numbers of tumor-associated macrophages^29,32^, which correlate with increased lymphangiogenesis and LVI^33–35^, in tumors belonging to Black women. Although no studies have directly compared racial differences in LVI in BCA, a multinational study evaluating clinicopathologic characteristics of urothelial tumors in white and Asian patients showed a significant racial difference in the rate of LVI^36^. No prior studies have evaluated the impact of LVI, RS, and race in the same population. We, therefore, sought to assess whether LVI adds prognostic or predictive information to the 21-gene RS, and whether there is an association between LVI and race that may contribute to racial disparities in EBC.

## Results

### Patient characteristics

**Fig. 1:**
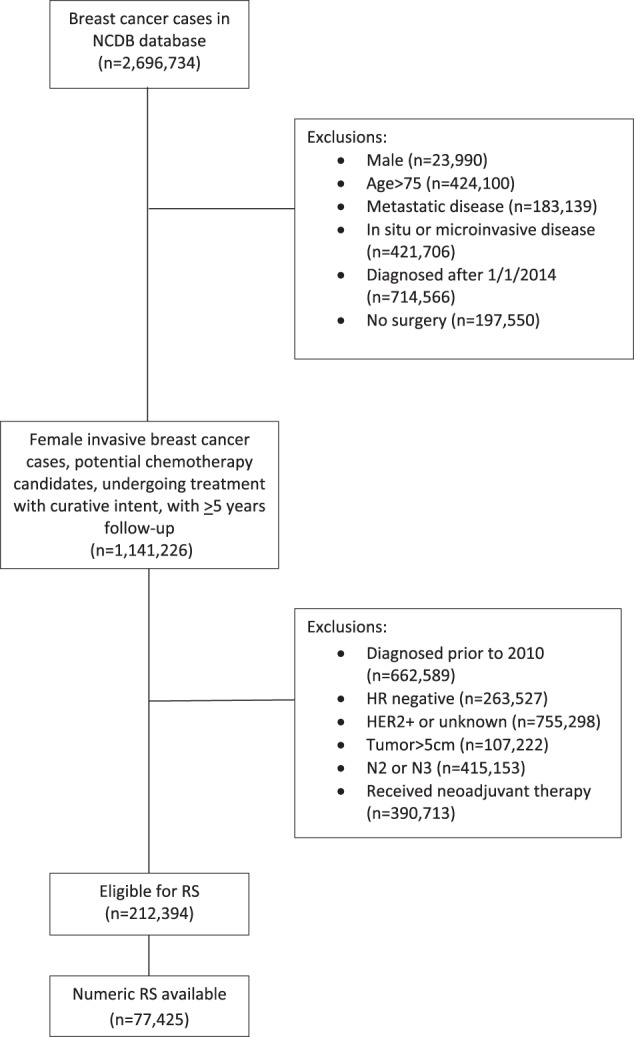
CONSORT flow diagram for patient selection. NCDB National Cancer Database, HR hormone receptor, N2, 4-9 involved axillary nodes, N3 10 or more involved axillary nodes, RS 21-gene recurrence score.

As shown in Fig. 1, of 2,696,734 women with breast cancer in the National Cancer Database (NCDB), 77,425 met inclusion criteria for this analysis, 65,018 of whom had no nodal involvement (N0), and 12,407 who had involvement of 1–3 axillary nodes (N+). Clinicopathologic and demographic characteristics of the study population are shown in Table 1. In all, 67,998 (87.8%) of patients were white, 5750 (7.4%) were Black, 3096 (4.0%) were members of other racial groups, and 581 (0.8%) had no racial information available. LVI was reported as present in 9856 (12.7%), absent in 59,305 (76.60%), and unknown in 8264 (10.67%).

**Table 1:** Patient characteristics (*n* = 77,425).

Characteristic	Number (%)
*Age (median, IQR)*	59 (50–65)
*Race*	
White	67998 (87.8%)
Black	5750 (7.4%)
Other	3096 (4.0%)
Unknown	581 (0.8%)
*LVI*	
Present	9856 (12.7%)
Absent	59,305 (76.6%)
Unknown	8264 (10.7%)
*Tumor size (mm)*	
0–20	59,396 (76.7%)
21–50	18,029 (23.3%)
*Lymph node involvement*	
0	65,018 (84.0%)
1–3^a^	12,407 (16.0%)
*Grade*	
1	21,069 (27.2%)
2	40,188 (51.9%)
3	12,077 (15.6%)
Unknown	4091 (5.3%)
*Recurrence score*	
0–10	17,390 (22.5%)
11–25	48,398 (62.5%)
26–100	11,637 (15.0%)
*Charlson–Deyo comorbidities*	
0	65,906 (85.1%)
1	9761 (12.6%)
2	1433 (1.8%)
3 or more	325 (0.4%)
*Median income quartile*	
<$38,000	9001 (11.6%)
$38,000–$47,999	15,080 (19.5%)
$48,000–$62,999	21,028 (27.2%)
>$63,000	32,146 (41.6%)
*Median educational level*^b^	
1 (17.6% or more)	8222 (10.6%)
2 (10.9%–17.5%)	16,236 (21.0%)
3 (6.3%–10.8%)	26,636 (34.5%)
4 (<6.3%)	26,190 (33.9%)

^a^Includes micrometastatic nodal involvement.

^b^% of adults > age 25 in patients’ zip code without high school diploma, expressed in quartiles.

### Association between LVI and other clinicopathologic features

As shown in Table 2, LVI was significantly associated with other poor prognostic clinicopathologic features, including axillary node metastasis (37.0% vs 12.2%), tumor size larger than 2 cm (37.1% vs. 21.0%), grade 3 histology (28.0% vs. 13.6%), and high RS of 26–100 (21.5% vs. 13.9%). These differences were substantial in magnitude and clinically relevant.

**Table 2:** Associations between LVI, race, and other clinicopathologic features.

	LVI present	LVI absent	LVI unknown	*P* value	White	Black	Other	*P* value
*Tumor size*				<0.0001				<0.0001
0–20 mm	6202 (62.9%)	46,843 (79.0%)	6351 (76.8%)		52,458 (77. 2%)	4192 (72.9%)	2308 (74.6%)	
21–50 mm	3654 (37.1%)	12,462 (21.01%)	1913 (23.15%)		15,540 (22.8%)	1558 (27.1%)	788 (25.4%)	
*Node involvement*				<0.0001				0.0253
Negative	6207 (63.0%)	52,094 (87.8%)	6717 (81.3%)		57,152 (84.0%)	4761 (82.8%)	2622 (84.7%)	
1–3+	3649 (37.0%)	7211 (12.2%)	1547 (18.7%)		10,846 (16.0%)	989 (17.2%)	474 (15.3%)	
*Tumor grade*				<0.0001				<0.0001
1	1222 (12.4%)	17,641 (29.8%)	2206 (26.7%)		18,964 (27.9%)	1251 (21.8%)	707 (22.8%)	
2	5407 (54.9%)	30,511 (51.5%)	4270 (51.7%)		35,196 (51.8%)	2973 (51.7%)	1712 (55.3%)	
3	2755 (28.0%)	8067 (13.6%)	1255 (15.2%)		10,187 (15.0%)	1252 (21.8%)	544 (17.6%)	
Unknown	472 (4.79%)	3086 (5.2%)	533 (6.5%)		3651 (5.4%)	274 (4.8%)	133 (4.3%)	
*Recurrence score*				<0.0001				<0.0001
0–10	1877 (19.0%)	13,675 (23.1%)	1838 (22.2%)		15,392 (22.6%)	1193 (20.8%)	682 (22.0%)	
11–25	5856 (59.4%)	37,405 (63.1%)	5137 (62.2%)		42,744 (62.9%)	3382 (58.8%)	1909 (61.7%)	
26–100	2123 (21.5%)	8225 (13.9%)	1289 (15.6%)		9862 (14.5%)	1175 (20.4%)	505 (16.3%)	

### Associations between race and other clinicopathologic features

Also shown in Table 2, Black race was associated with higher rates of axillary node metastasis (17.2% for Black women vs. 16.0% for white women vs. 15.3% for women of other races), tumor size larger than 2 cm (27.1% vs. 22.8% vs. 25.4%), grade 3 histology (21.8% vs. 15.0% vs. 17.6%), and high RS of 26–100 (20.4% vs. 14.5% vs. 16.3%). Although these differences were statistically significant, the absolute differences were marginal or modest. In contrast, LVI was slightly lower in Black women than in white women or women of other races (12.3% vs. 12.8% vs 13.2%, *p* = 0.0365).

### Association between LVI, race, and OS

Clinicopathologic features associated with poorer overall survival (OS) in univariate analysis were axillary nodal involvement, larger tumor size, higher grade, increasing RS, presence of LVI, and increased number of comorbidities (*p* < 0.0001 for all). Demographic factors associated with poorer OS in univariate analysis were Black race, older age, lower median educational level, and lower median income (*p* < 0.0001 for all) (Supplemental Table 1).

As shown in Table 3, factors associated with poor OS in multivariable analysis in the entire cohort of 77,425 patients included LVI (HR 1.24, *p* < 0.0001) and Black race (HR 1.24, *p* = 0.01). LVI was prognostic in the 65,018 patients with N0 (HR 1.37, *p* < 0.0001) but not in the 12,407 patients with N+ (HR 1.04, *p* = 0.66) disease, whereas Black race was prognostic in both N0 (HR 1.21, *p* = 0.009) and N+ (HR 1.37, *p* = 0.015) disease. Other factors associated with poorer OS included N+ disease (*p* < 0.0001), larger tumor size (*p* < 0.0001), grade 3 histology (*p* < 0.0001), higher RS (11–25 vs 0–10, *p* = 0.032, >26 vs 0–10, *p* < 0.0001), older age (*p* < 0.0001), lower median income (*p* = 0.0001), and greater number of comorbidities (*p* < 0.0001).

**Table 3:** Multivariate analysis of the association of clinicopathologic characteristics with OS.

	Entire cohort				Node Negative				Node Positive			
	(*n* = 75,602; deaths = 2810)				(*n* = 63,470; deaths = 2181)				(*n* = 12,132; deaths = 629)			
	HR	95% CI	*P* value		HR	95% CI	*P* value		HR	95% CI	*P* value	
*LVI*				0.0002				<0.0001				0.898
Present vs. absent	1.240	(1.119, 1.375)	<0.0001		1.372	(1.210, 1.555)	<0.0001		1.041	(0.872, 1.241)	0.658	
Unknown vs absent	1.088	(0.967, 1.225)	0.162		1.095	(0.956, 1.253)	0.191		1.032	(0.807, 1.320)	0.799	
*Tumor size (T2 vs T1)*	1.614	(1.491, 1.747)		<0.0001	1.619	(1.478, 1.773)		<0.0001	1.559	(1.327, 1.831)		<0.0001
*Recurrence score*								<0.0001				<0.0001
11–25 vs 0–10	1.119	(1.010, 1.240)	0.032	<0.0001	1.083	(0.964, 1.215)	0.178		1.291	(1.031, 1.618)	0.026	
26–100 vs 0–10	2.231	(1.944, 2.561)	<0.0001		2.035	(1.737, 2.383)	<0.0001		3.070	(2.301, 4.098)	<0.0001	
*Age at diagnosis*	1.050	(1.046, 1.055)		<0.0001	1.055	(1.05, 1.061)		<0.0001	1.034	(1.025, 1.044)		<0.0001
*Race*				<0.0001				0.001				0.004
Black vs white	1.243	(1.097, 1.408)	0.001		1.212	(1.050, 1.399)	0.009		1.368	(1.063, 1.761)	0.015	
Other vs white	0.643	(0.495, 0.835)	0.001		0.692	(0.522, 0.918)	0.011		0.451	(0.224, 0.909)	0.026	
*Chemotherapy (yes vs no)*	0.856	(0.772, 0.949)		0.003	0.910	(0.805, 1.029)		0.134	0.719	(0.592, 0.872)		0.001
*Grade*				<0.0001				<0.0001				0.001
2 vs 1	0.958	(0.869, 1.056)	0.387		0.915	(0.820, 1.020)	0.110		1.136	(0.916, 1.407)	0.245	
3 vs 1	1.346	(1.193, 1.519)	<0.0001		1.272	(1.109, 1.458)	0.001		1.640	(1.264, 2.129)	0.0002	
Unknown vs 1	1.062	(0.876, 1.288)	0.538		1.040	(0.838, 1.290)	0.721		1.145	(0.741, 1.767)	0.543	
*Nodes (N1 vs N0)*	1.365	(1.243, 1.500)		<0.0001	NA	NA	NA	NA	NA	NA	NA	NA
*Comorbidity*				<0.0001				<0.0001				<0.0001
1 vs 0	1.654	(1.508, 1.814)	<0.0001		1.728	(1.557, 1.918)	<0.0001		1.440	(1.178, 1.761)	0.0004	
2 vs 0	2.270	(1.904, 2.706)	<0.0001		2.368	(1.943, 2.886)	<0.0001		1.973	(1.343, 2.899)	0.001	
3 or more vs 0	4.004	(3.020, 5.310)	<0.0001		4.068	(2.878, 5.751)	<0.0001		3.773	(2.310, 6.161)	<0.0001	

### LVI, categorical RS, and overall survival in N0 disease

Among 65,018 patients with N0 disease, in whom LVI was prognostic, the presence of LVI was associated with worse OS in those with RS 11–25 [HR 1.31 (95% CI 1.09, 1.57)] and 26–100 [HR 1.58 (95% CI 1.30, 1.93)], but not in patients with RS 0–10 [HR 1.1 (95% CI 0.77, 1.53)], although there was no statistical interaction between LVI and RS (interaction *p* = 0.35) (Supplemental Table 2).

### Chemotherapy benefit

Receipt of chemotherapy was associated with improved OS in the entire cohort (*p* = 0.003) and in N+ patients (*p* = 0.001), but not in N0 patients (*p* = 0.134). There was no interaction between LVI and chemotherapy benefit in the 39,402 patients with N0 disease and an RS 11–25 (interaction *p* = 0.46) (Supplemental Table 3).

### Comparison of patients who did and did not undergo RS assay

Of 212,394 patients in the NCDB database eligible for genomic analysis, only 77,425 (36.5%) had numeric RS available. We compared characteristics of this group with the 119,321 patients who did not undergo RS analysis. Receipt of RS correlated with N0 disease, grade 2 histology, and absence of comorbidities (*p* < 0.0001), and was inversely associated with median educational and income levels (*p* < 0.0001). Both presence of LVI and Black race were associated with lower receipt of RS (12.7% vs 14.2% for LVI; 7.4% vs 9.2% for Black race, *p* < 0.0001). Use of RS was associated with lower likelihood of receiving chemotherapy (26.0% vs 34.8%, *p* < 0.0001) (Supplemental Table 4). Among women who did not have genomic testing, LVI was seen more frequently in non-white women than in white women (13.9% for white women, vs. 15.7% for Black women, vs. 17.1% for women of other races, *p* < 0.0001); however, the difference in frequency of LVI between white and Black women, whereas statistically significant, was numerically small in magnitude. Multivariable analysis of OS in women who did not undergo RS assay showed that LVI was associated with poorer OS in the entire cohort (HR = 1.21, *p* < 0.0001), and in both N0 (HR = 1.15, *p* = 0.008) and N+ patients (HR = 1.22, *p* < 0.00001). Black race was associated with poorer OS in the entire cohort (HS = 1.14, *p* = 0.0002) and in N+ (HR = 1.26, *p* < 0.0001) women only (Supplemental Table 5).

## Discussion

We evaluated the association between LVI, race, and the 21-gene RS in 77,425 women with ER-positive, HER2-positive EBC with 0–3 positive axillary nodes. We confirmed prior reports that LVI is associated with a worse prognosis in N0 disease^1,3,5,6^, and that Black race is associated with a worse prognosis^20,23,24^, but report for the first time no association between Black race and the presence of LVI. We also report for the first time that LVI adds prognostic information to the 21-gene RS, but not predictive information for chemotherapy benefit in those with a mid-range RS of 11–25.

Three previous studies have evaluated the correlation between LVI and the 21-gene RS in ER+ N0 EBC. Mutai et al.^16^ and Tan et al.^37^ evaluated 657 and 58 tumor samples, respectively, and reported no association between LVI and numeric RS. Mutai et al also reported that the presence of LVI was associated with poorer OS in patients with intermediate RS, but not in patients with low or high RS. In contrast, Turashvili et al.^38^ evaluated 2326 patients with EBC, reported an association of LVI with increasing RS, and found a trend toward greater likelihood of locoregional recurrence in patients with LVI (HR 1.81, 95% CI 0.96, 3.42, *p* = 0.06) after adjustment for RS.

Prior to the publication of the randomized TAILORx data^13^, the optimal management for patients with N0 ER+ BCA and intermediate RS was unclear, and recommendations for adjuvant chemotherapy in this group of patients were often based on clinicopathologic criteria. Chen et al.^39^ showed that the presence of LVI was significantly associated with receipt of chemotherapy in patients with intermediate RS diagnosed before 2013. Our study, although not randomized, did not detect a chemotherapy benefit in patients with intermediate RS and LVI who received adjuvant chemotherapy. In contrast, a retrospective evaluation of patients enrolled on two International Breast Cancer Study Group trials of chemoendocrine therapy in N0 EBC found that the benefit of ovarian suppression with goserelin in premenopausal patients with ER+ disease was exclusively confined to patients with LVI^40^. In addition, analysis of the BIG 1–98 trial showed that patients with LVI had greater than average benefit from letrozole compared with tamoxifen^41^. These findings are consistent with results of the SOFT/TEXT trials, indicating greater benefit from ovarian function suppression plus an aromatase inhibitor compared with tamoxifen in those at higher recurrence risk^42^.

Multiple factors contribute to poorer prognoses for Black breast cancer patients, including disparities in access to care^17,18^, later stage at diagnosis^43,44^, higher incidence of triple-negative disease^45^, and decreased adherence to chemotherapy and endocrine therapy regimens^46,47^. However, four clinical trials, which collectively included 26,138 patients, 2,370 (9%) of whom were characterized as Black (either by self-reported race, or by ancestry-informative genetic markers of African ancestry) have shown poorer outcomes for Black women with HR+ EBC^20–23^, despite similar antineoplastic therapy, indicating that differences in tumor biology in HR+ EBC also play a role. This disparity is hypothesized to be at least partially due to racial differences in the tumor microenvironment^24,25^. Although Black women have higher rates of obesity, which has been associated with higher recurrence rates in HR+ HER2− disease^48,49^, evidence suggests that this racial disparity is not explained by obesity alone^22,50^. Our analysis, using real-world data from NCDB, indicates that the racial disparity in outcome of ER+ EBC is not explained by a greater incidence of LVI, or by substantially and clinically relevant differences in the 21-gene RS.

Our study has several strengths and limitations, which are inherent in the use of NCDB data. Strengths include the large sample size, utilizing real-world data from CoC-accredited institutions, and representing the largest analysis evaluating the prognostic information provided by LVI, with a sufficient sample size to determine an association between LVI and Black race. Limitations include lack of information regarding cancer recurrence, both distant and locoregional, relatively short duration of follow-up, and relative under-representation of Black women in the EBC NCDB dataset^43^. An additional important limitation of our study is that the NCDB relies on local assessment of LVI, and does not distinguish between focal and extensive LVI. Prior reports indicate that up to 26% of local pathologists’ assessments of LVI are altered after pathologic review by an experienced breast pathologist^51^, and that extent of LVI correlates with poorer outcomes^6^. In addition, 10.67% of our cohort had unknown LVI status.

Our study is also limited by real-world biases in the ordering of RS. Comparison between our cohort and patients in the NCDB database who did not undergo RS assay show that patients in whom RS was ordered were more likely to have intermediate-grade tumors, N0 disease, and no comorbidities, confirming previously reported clinician tendencies to order RS in a state of clinical equipoise^52,53^, and in patients felt to be able to tolerate chemotherapy^53^. Lower receipt of RS in patients with LVI may reflect clinician bias in favor of administering chemotherapy to patients with LVI, a known poor prognostic factor. Black women were also somewhat under-represented in our cohort, compared with patients who did not receive RS. Previous studies evaluating racial bias in receipt of genomic testing have shown conflicting results. One study utilizing NCDB data showed that Black women were less likely than white women to undergo RS assay, and more likely to have testing ordered in guideline-discordant scenarios^54^. However, a more recent study, utilizing data from the Georgia Cancer Registry, found no racial differences in receipt of RS, but found a 1 year lag in uptake of testing in Black women^55^.

In summary, our data demonstrated that LVI adds prognostic information for OS in N0 ER+ HER2-BCA, with RS 11–100, but found no association between LVI and Black race in women undergoing RS assay. Despite the poorer prognosis associated with LVI, no chemotherapy benefit was seen in patient with RS 11–25 and LVI. Therefore the presence of LVI should not influence chemotherapy decision-making in patients with intermediate RS.

## Methods

### Case selection

We utilized a data set derived from the 2005–2016 National Cancer Database (NCDB). NCDB is a nationwide, facility-based database jointly sponsored by the American Cancer Society and the American College of Surgeons Commission on Cancer (CoC). The NCDB contains data collected on over 34,000,000 cancer cases from over 1500 CoC-accredited hospitals, representing over 70% of newly diagnosed cancers^56^, and 80% of newly diagnosed breast cancers in the United States^57^. The NCDB Participant User File (PUF) is a HIPAA-compliant data file, which is made available to investigators from CoC-accredited cancer programs who complete an application process. The data used in the study are derived from a de-identified NCDB file. The American College of Surgeons and the Commission on Cancer have not verified and are not responsible for the analytic or statistical methodology employed, or the conclusions drawn from these data by the investigator. The NCDB PUF contains de-identified patient data, including demographic information, tumor site, and pathology data, first course of treatment, and mortality. The NCDB PUF does not contain information on recurrence. The NCDB began collecting information on both LVI and on results of gene expression assays in 2010.

The study population included women age 75 and under-diagnosed between 1 January 2010 and 1 January 2014 with estrogen receptor-positive (ER+) HER2− BCA, measuring up to 5 cm, with 0–3 pathologically involved axillary nodes, treated with definitive surgery as first treatment, and with numeric RS available. Demographic information obtained included age, race, estimated annual household income and educational attainment. Race was classified as white, Black, other (including all other racial codes captured by the NCDB) or unknown. Clinical characteristics included tumor size in mm, histologic grade, axillary node involvement, LVI, and numeric RS. Micrometastatic nodal involvement (pN1mi) was classified as node positive. LVI was characterized as present, absent, or unknown. RS was characterized as low, intermediate, or high using TAILORx cutpoints, where 0–10 was defined as low, 11–25 as intermediate, and 26–100 as high.

### Statistical analysis

Bivariate associations between categorical variables were examined using the chi-square test. Multivariate Cox proportional hazards model were used to assess the association of LVI, chemotherapy, and RS on OS, while adjusting age, race, tumor size, grade, LN status, Charlson–Deyo comorbidity score, median income, and education level. The estimated HR for each variable in the model, along with its 95% CI, was reported. All tests are two-sided with a significance level ≤5%. Proportionality assumption was examined, and no violation was detected. All analyses were conducted using SAS 9.4 (SAS Institute Inc., Cary, NC, USA).

### Ethics

NCDB PUF data are de-identified, and compliant with HIPAA. Hospitals, health-care providers, and patients are not identified. Patient-informed consent is not obtained prior to institutional data submission to NCDB. As this study utilizes de-identified patient data, with no attempt made to contact or re-identify the subjects, it is deemed exempt from oversight by the Institutional Review Board of Albert Einstein College of Medicine.

### Reporting summary

Further information on research design is available in the Nature Research Reporting Summary linked to this article.

## Supplementary information

Supplemental Tables

Reporting Summary Checklist

## Data Availability

The data generated and analyzed during this study are described in the following data record: 10.6084/m9.figshare.13517210^58^. The data are stored in the National Cancer Database (NCDB) Participant User File (PUF) “NCDBPUF_Breast.0.2016.0.dat”. This is a HIPAA-compliant data file. The data use agreement between the researchers and the NCDB prohibits sharing of data, but the file can be made available to investigators from CoC-accredited cancer programs who complete an application process (CoC is the American College of Surgeons Commission on Cancer). Information regarding the application process is available at https://www.facs.org/quality-programs/cancer/ncdb/puf.

## Code availability

SAS 9.4 was used, and the code is available upon request.
