# Supplementary material for: Lymphovascular invasion, race, and the 21-gene recurrence score in early estrogen receptor-positive breast cancer
Source: NPJ Breast Cancer. 2021 Mar 1;7:20. doi: 10.1038/s41523-021-00231-x (PMC7921089; doi:10.1038/s41523-021-00231-x)
Supplement: Supplementary file 1 — Supplemental Tables [file 41523_2021_231_MOESM1_ESM.pdf]

Supplemental Table 1

Univariate analysis of association of clinicopathologic characteristics with OS

Characteristic	Number (%)		P value
	Alive	Dead	
Age (median) (IQR)	58 (50-65)	64 (57-70)	<0.0001
Race			<0.0001
White	65,514 (96.4%)	2484 (3.6%)	
Black	5439 (94.6%)	311 (5.4%)	
Other	3038(98.1%)	58 (2.0%)	
Unknown	564 (97.1%)	17 (2.9%)	
LVI			<0.0001
Present	9332 (94.7%)	524 (5.3%)	
Absent	57,286 (96.6%)	2019 (3.4%)	
Unknown	7937 (96.0%)	327(4.0%)	
Tumor size (mm)			<0.0001
0-20	57,569 (96.9%)	1827 (3.1%)	
21-50	16,986 (94.2%)	1043 (5.8%)	
Lymph node involvement			<0.0001
0	62,789 (96.6%)	2229 (3.4%)	
1-3*	11,766 (94.8%)	641 (5.2%)	
Grade			<0.0001
1	20,430 (97.0%)	639 (3.0%)	
2	38,839 (96.6%)	1349 (3.4%)	
3	11,322 (93.8%)	755 (6.2)	
Unknown	3964 (97.0%)	127 (3.0%)	
Recurrence Score			<0.0001
0-10	16,883 (97.1%)	507 (2.9%)	
11-25	46,865 (96.8%)	1533 (3.2%)	
26-100	10,807 (92.9%)	830 (7.1%)	
Charlson-Deyo Comorbidities			<0.0001
0	63,840 (98.9%)	2066 (3.1%)	
1	9149 (93.7%)	612 (6.3%)	
2	1292(90.2%)	141 (9.8%)	
3 or more	274 (84.3%)	51 (15.7%)	
Median income quartile			<0.0001
<$38,000	8513 (94.6%)	488 (5.4%)	
$38,000 - $47,999	14,425 (95.7%)	655 (4.3%)	
$48,000 - $62,999	20,237 (96.2%)	791 (3.8%)	
>$63,000	31,214 (97.1%)	932 (2.9%)	
Median educational level **			<0.0001
1 (17.6% or more)	7832 (95.3%)	390 (4.7%)	
2 (10.9% - 17.5%)	15,515 (95.6%)	721 (4.4%)	
3 (6.3% - 10.8%)	25,636 (96.2%)	1000 (3.8%)	
4 (<6.3%)	25,435 (97.1%)	755 (2.9%)	

* includes micrometastatic nodal involvement

**% of adults $\geq$ age 25 in patients' zip code without high school diploma, expressed in quartiles

Supplemental Table 2

Interaction between LVI and RS in node negative patients

(n=63470, number of deaths 2181)

	HR	95% CI	p-value
LVI			0.665
Present vs. absent, RS 0-10	1.089	(0.773, 1.532)	
Present vs. absent, RS 11-25	1.307	(1.086, 1.572)	
Present vs. absent, RS 26-100	1.583	(1.302, 1.925)	
Unknown vs absent, RS 0-10	1.136	(0.836, 1.543)	
Unknown vs absent, RS 11-25	1.050	(0.869, 1.269)	
Unknown vs absent, RS 26-100	1.153	(0.898, 1.480)	
RS			<0.0001
11-25 vs 0-10 in LVI absent	1.072	(0.942, 1.221)	
26-100 vs 0-10 in LVI absent	1.928	(1.621, 2.295)	
11-25 vs 0-10 in LVI present	1.287	(0.893, 1.855)	
26-100 vs 0-10 in LVI present	2.804	(1.922, 4.092)	
11-25 vs 0-10 in LVI unknown	0.991	(0.708, 1.389)	
26-100 vs 0-10 in LVI unknown	1.958	(1.338, 2.866)	
Interaction between LVI and RS			0.352

Supplemental Table 3

Interaction between LVI and chemotherapy in node negative patients with RS=11-25

(n=39402, number of deaths 1138)

	HR	95% CI	p-value
LVI			0.025
Present vs. absent, no chemotherapy	1.331	(1.082, 1.638)	
Present vs. absent, received chemotherapy	1.234	(0.813, 1.875)	
Unknown vs absent, no chemotherapy	0.999	(0.810, 1.233)	
Unknown vs absent, received chemotherapy	1.330	(0.852, 2.076)	
Chemotherapy			0.904
Yes vs no in LVI absent	0.988	(0.813, 1.201)	
Yes vs no in LVI present	0.916	(0.598, 1.403)	
Yes vs no in LVI unknown	1.315	(0.834, 2.074)	
Interaction between LVI and chemotherapy			0.46

Supplemental Table 4

Comparison of Characteristics of Patients in Whom RS Was vs. Was Not Evaluated

Characteristic	Number (%) RS done (n=77,425)	Number (%) RS not done (n=119,321)	P value
Age (median, IQR)	59 (50-65)	61 (52-68)	<0.0001
Race			<0.0001
White	67,998 (87.8%)	102,395 (85.8%)	
Black	5750 (7.4%)	10938 (9.2%)	
Other	3096 (4.0%)	5034 (4.2%)	
Unknown	581 (0.8%)	954 (0.8%)	
LVI			<0.0001
Present	9856 (12.7%)	16,969 (14.2%)	
Absent	59,305 (76.6%)	87,875 (73.6%)	
Unknown	8264 (10.7%)	14,477 (12.1%)	
Tumor size (mm)			<0.0001
0-20	59,396 (76.7%)	90,222 (75.6%)	
21-50	18,029 (23.3%)	29,099 (24.4%)	
Lymph node involvement			<0.0001
0	65,018 (84.0%)	87,088 (73.0%)	
1-3*	12,407 (16.0%)	32,233 (27.0%)	
Grade			<0.0001
1	21,069 (27.2%)	38,976 (32.7%)	
2	40,188 (51.9%)	54,255 (45.5%)	
3	12,077 (15.6%)	19,162 (16.1%)	
unknown	4091 (5.3%)	6928 (5.8%)	
Recurrence Score			
0-10	17,390 (22.5%)	NA	
11-25	48,398 (62.5%)	NA	
26-100	11,637 (15.0%)	NA	
Charlson-Deyo Comorbidities			<0.0001
0	65,906 (85.1%)	98,980 (83.0%)	
1	9761 (12.6%)	16,554 (13.9%)	
2	1433 (1.8%)	2968 (2.5%)	
3 or more	325 (0.4%)	819 (0.7%)	
Median income quartile			<0.0001
<$38,000	9001 (11.6%)	17,044 (14.3%)	
$38,000 - $47,999	15,080 (19.5%)	25,247 (21.2%)	
$48,000 - $62,999	21,028 (27.2%)	31,898 (26.8%)	
>$63,000	32,146 (41.6%)	44,881 (37.7%)	
Median educational level**			<0.0001
1 (17.6% or more)	8222 (10.6%)	16,282 (13.7%)	
2 (10.9% - 17.5%)	16,236 (21.0%)	28,200 (23.7%)	
3 (6.3% - 10.8%)	26,636 (34.5%)	39,607 (33.2%)	
4 (<6.3%)	26,190 (33.9%)	35,012 (29.4%)	

* includes micrometastatic nodal involvement

**% of adults > age 25 in patients' zip code without high school diploma, expressed in quartiles

Supplemental Table 5

Multivariate analysis of characteristics associated with OS in patients who did not undergo genomic testing

	Entire Cohort (n=116,121; deaths=7725)				Node Negative (n=84,531; deaths=5030)				Node Positive (n=31,590; deaths=2695)			
	HR	95% CI	P value		HR	95% CI	P value		HR	95%CI	P-value	
LVI				<0.0001				0.026				<0.0001
Present vs. absent	1.208	(1.134, 1.287)	<0.0001		1.150	(1.036, 1.275)	<0.0001		1.219	(1.122, 1.324)	<0.0001	
Unknown vs absent	1.003	(0.935, 1.077)	0.923		1.039	(0.954, 1.132)	0.383		0.946	(0.836, 1.070)	0.377	
Tumor size (T2 vs T1)	1.582	(1.500, 1.667)		<0.0001	1.516	(1.412, 1.628)		<0.0001	1.569	(1.449, 1.698)		<0.0001
Age at Diagnosis	1.046	(1.043, 1.049)		<0.0001	1.058	(1.054, 1.062)		<0.0001	1.030	(1.025, 1.034)		<0.0001
Race				<0.0001				<0.0001				<0.0001
Black vs White	1.143	(1.064, 1.227)	0.0002		1.062	(0.967, 1.166)	0.210		1.258	(1.127, 1.405)	<0.0001	
Other vs White	0.679	(0.586, 0.787)	<0.0001		0.602	(0.493, 0.735)	<0.0001		0.799	(0.640, 0.997)	0.047	
Chemotherapy (Yes vs No)	0.803	(0.753, 0.856)		<0.0001	1.045	(1.052, 1.245)		0.002	0.719	(0.592, 0.872)		<0.0001
Grade				<0.0001				<0.0001				<0.0001
2 vs 1	1.177	(1.112, 1.246)	<0.0001		1.146	(1.074, 1.224)	<0.0001		1.265	(1.122, 1.426)	<0.0001	
3 vs 1	1.880	(1.754,2.016)	<0.0001		1.621	(1.482, 1.772)	<0.0001		2.205	(1.941, 2.505)	<0.0001	
unknown vs 1	1.314	(1.181,1.461)	<0.0001		1.248	(1.100, 1.417)	0.0001		1.489	(1.218, 1.819)	<0.0001	
Node involvement (N1 vs N0)	1.410	(1.327,1.497)		<0.0001	NA	NA	NA	NA	NA	NA	NA	NA
Comorbidity				<0.0001				<0.0001				<0.0001
1 vs 0	1.686	(1.595,1.782)	<0.0001		1.793	(1.676, 1.918)	<0.0001		1.521	(1.381, 1.675)	<0.0001	
2 vs 0	2.970	(2.718,3.245)	<0.0001		3.393	(3.053, 3.771)	<0.0001		2.240	(1.902, 2.638)	<0.0001	
3 or more vs 0	4.501	(3.936,5.147)	<0.0001		5.204	(4.437, 6.103)	<0.0001		3.314	(2.586, 4.247)	<0.0001	
